# Phosphatidylserine-decorated delivery platform helps alleviate acute lung injury via potentiating macrophage targeting

**DOI:** 10.1016/j.jlr.2025.100799

**Published:** 2025-04-10

**Authors:** Yue Li, Hu Li, Zhiwei Hu, Yayue Zhang, Xuran Ding, Xinjie Huang, Yabing Hua, Lin Sun, Ye Li, Ziming Zhao, Yuan He

**Affiliations:** 1 School of Medical Technology, Xuzhou Medical University, Xuzhou, Jiangsu, China; 2 School of Pharmacy, Xuzhou Medical University, Xuzhou, Jiangsu, China; 3 The Second Clinical Medical School, Xuzhou Medical University, Xuzhou, Jiangsu, China; 4 Department of Zhuhai Campus of Zunyi Medical University, Zhuhai, China; 5 The Third Affiliated Hospital of Sun Yat-sen University, Guangzhou, Guangdong, China

**Keywords:** phosphatidylserine, decoration, macrophage, delivery platform, acute lung injury, apoptotic cell

## Abstract

Acute lung injury (ALI) is a life-threatening inflammatory disease with high morbidity and mortality. It is urgent to develop more effective therapeutic strategies against ALI. Phosphatidylserine (PtdSer) expressed on the surface of apoptotic cells not only allows for macrophage binding and recognition but also drives anti-inflammatory signaling within the macrophage. In this study, we designed an apoptotic cell-mimicry nanoparticle by decorating synthetic PtdSer on the outer face of nanoparticles. The results indicated that PtdSer-decorated poly(lactic-co-glycolic acid) nanoparticles (PSNPs) showed anti-inflammatory properties and increased macrophage phagocytosis in relative to the nondecorated poly(lactic-co-glycolic acid nanoparticles. Dexamethasone-loaded PSNPs exhibited superior anti-inflammatory activity on macrophages in vitro. In vivo studies also showed that PtdSer decoration increased the accumulation of nanoparticles in lung macrophages after pulmonary administration. Accumulation of dexamethasone-loaded PSNPs in lung macrophages effectively reduced inflammation in inflamed lungs and further alleviated ALI syndromes. In conclusion, PtdSer decoration not only endows the anti-inflammatory function to nanocarriers but also potentiates its macrophage targeting in the inflamed microenvironment, which offers an ideal drug delivery platform for ALI therapy.

Acute lung injury (ALI) is a common, life-threatening critical public health problem that leads to significant morbidity and mortality (1). Although significant efforts have been made in understanding the pathophysiology of ALI and in developing pharmacological treatments, clinical trials revealed that these pharmacological therapies are not as effective as expected (2). For instance, due to the difficulty of the drugs to reach the injured lungs, high-dose corticosteroids are commonly used to achieve satisfactory therapeutic efficacy, which increases the risk of systemic adverse reactions (3, 4). Therefore, it is urgent to develop more effective treatments for the direct delivery of drugs in the injured lungs.

ALI is characterized by an overwhelming lung parenchymal inflammation. During the pathogenesis of ALI, the innate immune cells release many proinflammatory cytokines, which give rise to an inflammation cascade (5). Macrophages, the important components of innate immunity, have been proven to play a crucial role in the development of ALI (6, 7). Since ALI primarily occurs in lung tissues, pulmonary delivery is considered to be a preferable route for drug delivery. However, the receptors of glucocorticoid are widely expressed in most cell types of the inflamed lungs, and the free glucocorticoid after pulmonary delivery may lead to other side effects (8, 9). Theoretically, the precise targeting delivery of drugs to lung macrophages should increase the drug concentration at the inflammatory lungs, and regulating the function of activated macrophages in the injured lungs might be a promising therapeutic strategy against ALI (10).

In recent decades, various macrophage-targeted nanoparticles (NPs) have been developed for therapy and diagnosis (11, 12). However, the proinflammatory effects of exogenous NPs have been reported in the literature, arousing our attention to side effects of nanotechnological-based strategies. Different from exogenous irritants, apoptotic cell clearance (also known as “efferocytosis”) is a tightly regulated physiologic process without an inflammatory immune response (13, 14). This efferocytosis process is mediated by phosphatidylserine (PtdSer) on the surface of apoptotic cells, which not only allows for macrophage binding and recognition but also drives anti-inflammatory signaling within the macrophage (15, 16).

Based on the above, we designed an apoptotic cell-mimicry NP for ALI treatment by decorating synthetic PtdSer on the outer face of poly (lactic-co-glycolic acid) (PLGA) nanoparticles and termed them PSNPs (Fig. 1). Dexamethasone (DEM), a corticosteroid drug, was capsulated into the core of PSNPs for anti-inflammatory therapy. The targeting ability and therapeutic effect of DEM-loaded PSNPs (PSNPs@DEM) to lung macrophages was evaluated in a lipopolysaccharide (LPS)-induced ALI model after pulmonary administration. This work herein describes the use of PtdSer-decorated nanocarriers as a drug delivery platform for ALI therapy, which offers precise drug targeting to inflamed lungs and the potential to reduce off-target and systemic adverse effects.

**Fig. 1:**
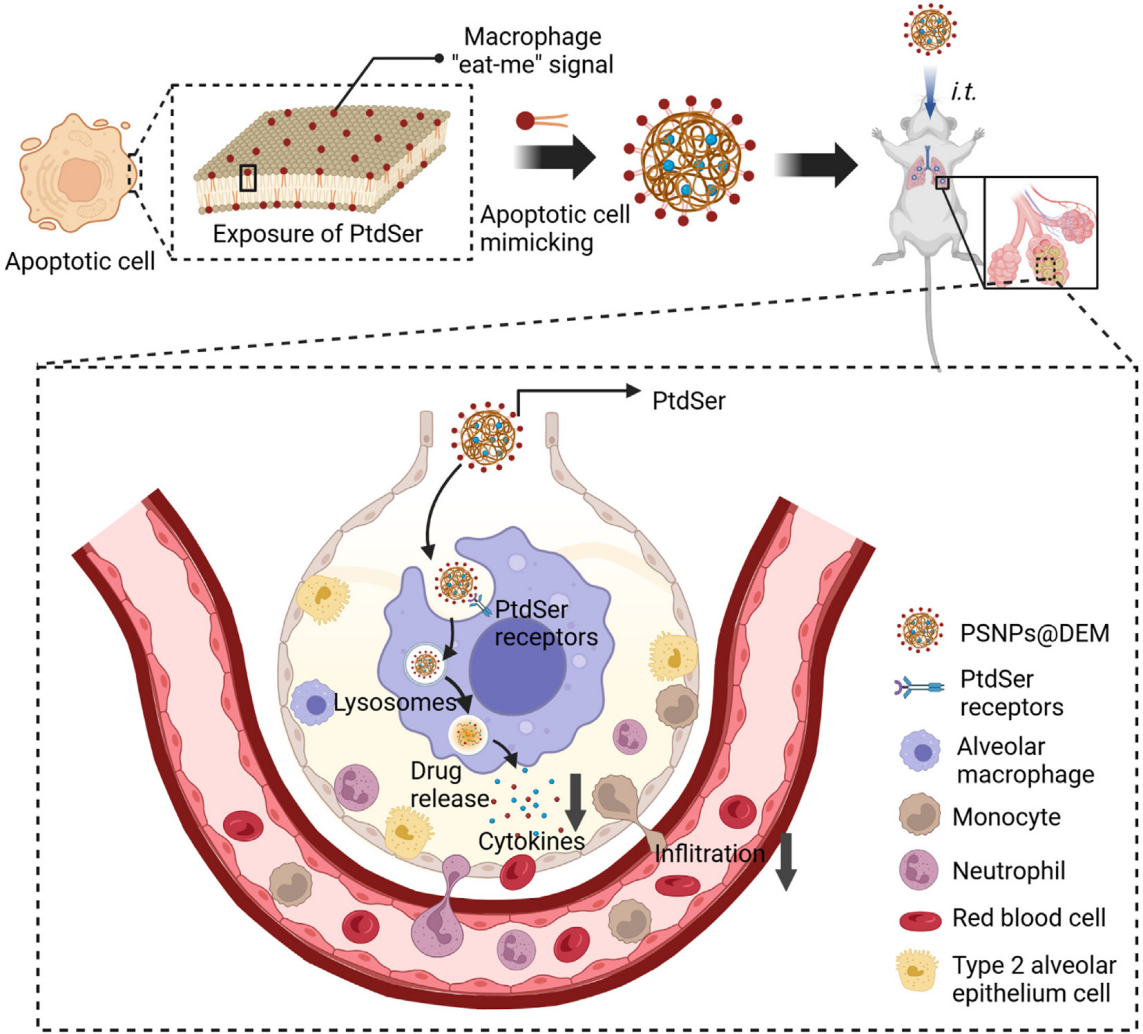
Schematic diagram showing the synthesis of the apoptotic cell-mimicking PSNPs@DEM and its modulatory effects on lung macrophages in the ALI mouse model (created with BioRender.com). ALI, acute lung injury; PSNP@DEM, dexamethasone-loaded PtdSer-decorated PLGA nanoparticle.

## Materials and Methods

### Materials

DEM was purchased from Rhawn Reagent (Shanghai, China). PLGA (lactic acid/glycolic acid 50/50, 14 kDa) was purchased from Xi’an Ruixi Biological Technology Co., Ltd. (Xi’an, China). Polyvinyl alcohol was obtained from Wing Hing Chemical Company Ltd. (Hong Kong, China). 1,2-dioleoyl-sn-glycero-3-phospho-L-serine sodium salt (DOPS) and 1,2-dipalmitoyl-sn-glycero-3-phosphocholine was obtained from Suzhou Highfine Biotech Co., Ltd. (Suzhou, China). LPS was purchased from Sigma (St. Louis, MO). Hoechst 33342, LysoTracker Red, ER-tracker Red, Golgi-tracker Red, Annexin V-FITC, and HBSS were purchased from Beyotime Biotechnology (Shanghai, China). 3,3′-dioctadecyloxacarbocyanine perchlorate (DiO), 1,1′-dioctadecyl-3,3,3′,3′- tetramethylindocarbocyanine perchlorate (DiI), and 1,1′-dioctadecyl-3,3,3′,3′-tetramethylindodicarbocyanine, 4-chlorobenzenesulfonate salt (DiD) were obtained from BBI Life Sciences Corporation (Shanghai, China). FITC anti-mouse F4/80 (Cat # 123107), annexin V (Cat # 640901), and biotin anti-mouse F4/80 antibodies (Cat # 123105) were purchased from BioLegend (San Diego). F4/80 monoclonal antibody (Cat # MF48000) was purchased from Invitrogen. Cy3-conjugated AffiniPure goat anti-rat IgG secondary antibody (Cat # GB21302) was purchased from Servicebio (Wuhan, China). Streptavidin magnetic beads (Cat # HY-K0208) were purchased from MedChemExpress (Shanghai, China). Mouse IL-1β ELISA Kit (Cat # EK201B), mouse TNF-α ELISA Kit (Cat # EK282), and mouse IL-6 ELISA Kit (Cat # EK206) were purchased from Multi Sciences (Hangzhou, China). All other chemicals and reagents were obtained from commercial sources and were of analytical grade.

### Cell culture

RAW 264.7 cells (ATCC, Manassas) were cultured in high-glucose DMEM (Gibco) supplemented with 10% FBS (Clark) and 100 U/ml/100 μg/ml penicillin-streptomycin (HyClone, Logan) in a 37°C incubator with 5% CO_2_.

### Animals

Male and female BALB/c mice (6–8 weeks) were purchased from Beijing Vital River Laboratory Animal Technology Co., Ltd. All relevant experimental protocols were approved by the Animal Research Ethics Committee of Xuzhou Medical University. All efforts were made to minimize animal suffering and to limit the number of animals used.

### Preparation and characterization of PSNPs

PLGA NP) were prepared by the antisolvent precipitation method (17, 18). Briefly, PLGA was dissolved in DMSO. Then, the organic solution was injected into PBS containing 0.1% polyvinyl alcohol under stirring. NPs were then acquired by centrifuging and resuspending to remove the organic solvent. For the preparation of PSNPs with different PtdSer decoration densities, DOPS were codissolved with PLGA in DMSO at different ratios, such as low PtdSer decoration density NPs (2% PtdSer, L-PSNPs), moderate PtdSer decoration density NPs (5% PtdSer, M-PSNPs), and high PtdSer decoration density NPs (10% PtdSer, H-PSNPs). DEM-loaded nanocarriers were prepared by codissolving DEM, PLGA, and DOPS in DMSO, and then the organic phase was injected into water phase as described above. To prepare DiO-labeled, DiI-labeled, and DiD-labeled nanocarriers, these fluorescent dyes (DiO, DiI, and DiD) were added into the organic phase, respectively, and all other procedures were the same as those described above.

Then, the particle size, polydispersity index, and *zeta* potential of the nanocarriers were measured by using a nanobrook Omni (Brookhaven Instruments). The stability was studied by monitoring the particle size over 7 days of storage at 4°C. For the morphology study, the samples were negatively stained with 2% phosphotungstic acid and photographed using a transmission electron microscope (Tecnai G2 Spirit Twin, FEI). The encapsulation efficiency (EE%) and drug loading capacity (DL%) of DEM-loaded nanocarriers were determined by using an HPLC (Hitachi Primaide™ HPLC system). The EE% and DL% were calculated according to the equations below:

$$EE\;\left(\%\right)=\frac{total\;drug\;amount-free\;drug\;amount}{total\;drug\;amount}$$

$$DL\;\left(\%\right)=\frac{the\;amount\;of\;drug\;loaded}{drug\;amount+excipients\;amount}$$

### Quantification of PtdSer on PSNPs

After the preparation of PSNPs with different decoration densities, the amount of PtdSer exposed on the nanocarriers was quantified using the annexin V-FITC, which can bind to PtdSer and label them on the nanocarriers (19). Briefly, the prepared PSNPs were incubated with annexin V-FITC, then the unbound annexin-FITC was removed by centrifugation, and the precipitates were suspended in PBS. The fluorescence intensity was measured at 525 nm using a microplate reader (BioTek Instruments).

### In vitro drug release study

In vitro drug release study was investigated by the dialysis method in PBS, (pH 7.4). Briefly, 1 ml of each formulation containing 400 μg/ml DEM was added to a dialysis bag (Mw 8000–14000), and then placed in 40 ml of PBS release medium under stirring at 100 rpm and 37 ± 0.5°C. At predetermined time points, a 1 ml sample was taken and then replaced immediately with the same volume of prewarmed fresh PBS. The DEM concentration in the collected samples was quantified by a UV absorption wavelength of 240 nm. Then the cumulative release rate of DEM at different times was calculated.

### In vitro macrophage phagocytosis study

The cytotoxicity of nanocarriers was assessed by the cell counting kit-8 (CCK-8) assay. RAW 264.7 cells were treated with nanocarriers at the DEM concentrations of 5, 10, 20, 50, and 100 μg/ml, along with an untreated group. After 24 h incubation, the nanocarriers were removed and CCK-8 reagent was added according to the manufacturer's instructions. Then, absorbance was measured using a microplate reader at 450 nm.

Macrophage phagocytosis of NPs and three densities of PSNPs (L-PSNPs, M-PSNPs, and H-PSNPs) were carried out by flow cytometry (Agilent Novocyte Cytometer). Briefly, RAW 264.7 cells were treated with 1 ml of DiO-labeled nanocarriers (diluted with cell culture medium to obtain a DiO concentration of 5 μg/ml) at 37°C for 2 h, and then cells were trypsinized and centrifuged. The cell precipitates were resuspended in PBS. The mean fluorescence intensity of the cells was measured by flow cytometry.

To confirm the role of PtdSer in phagocytosis, annexin V was used to block PtdSer. Annexin V was preincubated with DiI-labeled PSNPs for PtdSer blocking, and then the blocked and unblocked nanocarriers were added to RAW 264.7 cells. The macrophage phagocytosis of nanocarriers with or without annexin V blocking was compared by observing the fluorescence intensity under confocal laser scanning microscopy (CLSM, Leica TCS SP8, Germany) and quantitatively determined by flow cytometry.

### Intracellular trafficking pathway within cells

The intracellular trafficking pathways of NPs and PSNPs within RAW 264.7 cells were studied via colocalization. Cells were incubated with DiO-labeled NPs and PSNPs for 1 h at 37°C. After incubation, nanocarriers were removed. Then, subcellular organelles, including lysosomes, the Golgi apparatus, and the endoplasmic reticulum (ER), were stained with 1 μM Lysotracker Red, 5 μM Golgi Tracker Red, and 1 μM ER Tracker Red, respectively. The nuclei were stained and then imaged by CLSM. The obtained images were further used for colocalization analysis. The colocalization values (Pearson's correlation (R)) were calculated by ImageJ software.

### Real-time disintegration monitoring by the FRET technique

To monitor the disintegration of NPs and PSNPs, the Förster resonance energy transfer (FRET) technique was applied. First, the NPs and PSNPs with the FRET signals were fabricated by adding the FRET pair DiO and DiI to the PLGA NPs at a molecular ratio of 1:1. The FRET signals of the nanocarriers were verified by fluorescence spectrum scanning with a microplate reader. In this experiment, 0.2% 1,2-dipalmitoyl-sn-glycero-3-phosphocholine was employed as the simulated lung fluid (SLF), since it is the predominant constituent of lipids in pulmonary surfactant (20, 21, 22). The nanocarriers with FRET signals were then incubated with SLF and RAW 264.7 cells. For incubation in cells, the nanocarriers were incubated with RAW cells for 1 h, then removed and replaced with fresh HBSS, and the cells were further incubated for different durations (0 h, 1 h, 2 h, and 4 h). The absorbance of the cells in the plates was measured by using a microplate reader at an excitation wavelength of 470 nm. The FRET ratio was then calculated by using the equation FRET ratio = *I*_*acceptor*_/(*I*_*donor*_ + *I*_*acceptor*_). Furthermore, the FRET signals were also observed directly by the CLSM.

### In vitro anti-inflammatory effect determination

RAW 264.7 cells were pretreated with 100 ng/ml of LPS for 1 h to stimulate inflammation, and then DEM-containing nanocarriers were added to each group for 24 h of treatment. After incubation, the treated cells were collected for quantitative real-time PCR (qPCR) assay. Total RNA was extracted using TRIzol reagent (Invitrogen), and the extracted RNA was reverse transcribed into cDNA with PrimeScript™ RT Master Mix (TaKaRa Bio, China). qPCR assays were performed with Universal SYBR Green Fast qPCR Mix (ABclonal, China) using the LightCycler® 480 System (Roche) (primers used in this study are listed in supplemental Table S1).

### Inflammatory cytokine detection

Cell culture supernatant was collected at 24 h posttreatment, and cytokines such as interleukin-6 (IL-6), interleukin-1β (IL-1β), and tumor necrosis factor-α (TNF-α) in the supernatant from in vitro cultured macrophages were quantified using ELISA kits following the manufacturer’s instructions.

### In vivo targeting delivery to lung macrophages

Before the experiment, ALI model mice were established according to previous reports (23). Male BALB/c mice were anesthetized and then intratracheally administered with LPS solution (8 mg/kg). After 4 h, ALI mice were treated with DiO-labeled nanocarriers by intratracheal administration. The animals were then sacrificed 4 h after administration, and the lung tissues were harvested and washed by rinsing the whole lung with PBS for further analysis. The left upper lobe of the lung was collected and cryosectioned at a thickness of 15 μm. The slides with tissue sections were then fixed and blocked with 1% BSA. Subsequently, F4/80 primary antibody incubation was carried out for macrophage labeling, followed by Cy3-conjugated secondary antibody incubation. Images were acquired by observing tissue samples under CLSM.

Then, phagocytosis of macrophages in the lung was quantitatively determined by flow cytometry. Briefly, the lung tissues of mice after administration of DiD-labeled nanocarriers for 4 h were harvested and chopped with scissors into small pieces. Tissue fragments were immersed in the digestion solution (2.5 mg/ml collagenase type IV) at 37°C for 2 h. Following incubation, digested lung samples were squeezed and filtered to prepare the single-cell suspension. FITC anti-mouse F4/80 antibodies were used to label lung macrophages. Then, cells were washed and used for flow cytometry. The acquired data were analyzed with FlowJo, version 10.1 (Tree Star). The fluorescence intensity of nanocarriers was analyzed in F4/80^+^ macrophages.

### Therapeutic effect of PSNPs@DEM in ALI mice

The ALI mouse model was generated as described above. Twenty-four male mice were randomly divided into four groups with six animals in each group, including healthy control, LPS-induced ALI, ALI+NPs@DEM, and ALI+PSNPs@DEM groups (at the DEM concentration of 2 mg/kg). The mice from the healthy group received the same treatment, but PBS was used instead of LPS and nanocarriers. The nanocarriers were administered to the mice after 4 h of LPS pretreatment. Then, 24 h after administration, bronchoalveolar lavage fluid (BALF) and lung tissues were collected. The BALF was then centrifuged at 500 *g* at 4°C for 7 min to obtain cell pellets and supernatants. The obtained supernatant was used for the determination of the total protein concentration with a BCA protein kit (Beyotime, Biotech). The separated cell pellets in BALF were resuspended in flow cytometry staining buffer and stained with FITC anti-mouse F4/80 antibody to determine the total cell count and percentage of macrophages by using a flow cytometer.

Then, the relative mRNA levels of inflammatory cytokines in lung tissues and lung macrophages were analyzed, respectively. For the analysis of lung tissues, whole lung tissues were homogenized, and then total RNA was extracted using TRIzol reagent and collected for qPCR analysis. To determine the mRNA levels in macrophages, macrophages of lung tissues were isolated by using magnetic-activated cell sorting beads. Briefly, the lung tissues of the mice after treatment were harvested and digested to prepare the single-cell suspension. Then, biotin anti-mouse F4/80 antibodies were used to label lung macrophages. Biotin-positive lung cells were extracted from whole lung cells by streptavidin magnetic beads and a permanent magnet. The F4/80^+^ macrophages sorted from lung tissues were collected for qPCR analysis. To test the applicability of the treatment, 24 female mice were also used to examine the therapeutic effect of PSNPs@DEM. The relative mRNA levels of inflammatory cytokines in lung tissues of female ALI mice were analyzed after treatment.

Furthermore, the histological examination was conducted by H&E staining of lung sections and observation via optical microscopy. The degree of lung injury was then assessed by scoring the five histological features of the lungs, including interstitial neutrophils, alveolar neutrophils, hyaline membranes, alveolar septal thickening, and proteinaceous debris (24).

### Statistical analysis

In this study, at least three independent experiments were performed, and all the data are shown as the means ± SDs. Comparisons for two groups were performed using unpaired two-tailed Student's *t* tests, and comparisons of more than two groups were performed using one-way ANOVA with multiple comparison tests. Differences were considered statistically significant at ∗*P* < 0.05.

## Results

### Preparation and characterization of PtdSer-decorated nanocarriers

**Fig. 2:**
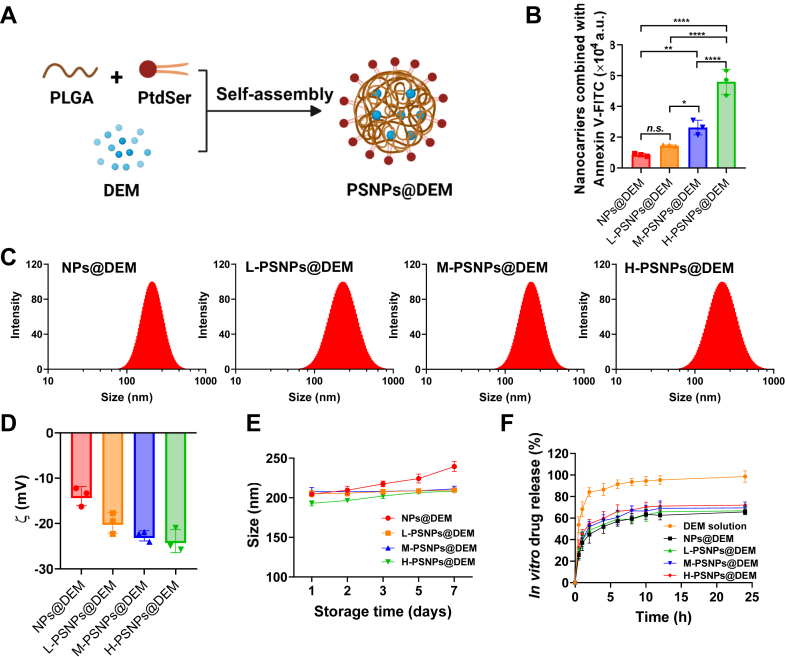
A: Schematic diagram showing the preparation of DEM-loaded PSNPs (PSNPs@DEM). B: A microplate reader was used to examine the PtdSer that decorated on the surface of nanoparticles by annexin V-FITC (∗*P* < 0.05, ∗∗*P* < 0.01, and ∗∗∗∗*P* < 0.0001; n.*s*., not significant.) C: The size distribution of different nanoparticles. D: The *zeta potential* (ζ) values of different nanoparticles. E: The storage stability of nanoparticles at 4°C for 7 days (F) In vitro drug release profiles of DEM solution and DEM-loaded nanocarriers at pH 7.4. (n = 3). DEM, dexamethasone; PtdSer, phosphatidylserine.

To simulate the apoptotic cell surface, synthetic PtdSer was decorated on the outer face of PLGA NPs by an antisolvent precipitation method. Briefly, PtdSer was incorporated into the PLGA NPs through the insertion of the hydrophobic tail in the core and the hydrophilic domain of PtdSer exposed on the surface of the NP. The schematic structure of the PSNPs@DEM is illustrated in Figure 2A. To identify the optimal density of PtdSer decoration, nondecorated PLGA NPs loaded with DEM (NPs@DEM), and threePSNPs@DEM were prepared. Annexin V, a cellular protein, has a specific affinity for PtdSer exposed on the surface of apoptotic cells. With increasing amounts of PtdSer within PSNPs@DEM, the combined annexin V-FITC fluorescence intensity increased (Fig. 2B), revealing the successful decoration of PtdSer on the NPs.

The NPs@DEM and three PSNPs@DEM had an average size of approximately 200 nm (supplemental Table S1), with a uniform size distribution (Fig. 2C). Transmission electron microscope images revealed that both the NPs@DEM and three PSNPs@DEM had a spherical shape (supplemental Fig. S1). All of the nanocarriers had a negative surface charge. The *zeta* potential became more negative with increasing amounts of PtdSer within PSNPs@DEM (Fig. 2D). The EE% and DL% are also shown in supplemental Table S2. The prepared nanocarriers remained stable for 7 days (Fig. 2E). In vitro drug release profiles demonstrated that the release rates of DEM in NPs@DEM and three PSNPs@DEM groups were slower than that of the DEM solution group, exhibiting a sustained-release behavior. For the drug release of these different NPs, the release rate increased slightly as the amount of PtdSer added to the nanocarriers increased (Fig. 2F).

### PtdSer decoration enhanced phagocytosis of nanocarriers by macrophages

**Fig. 3:**
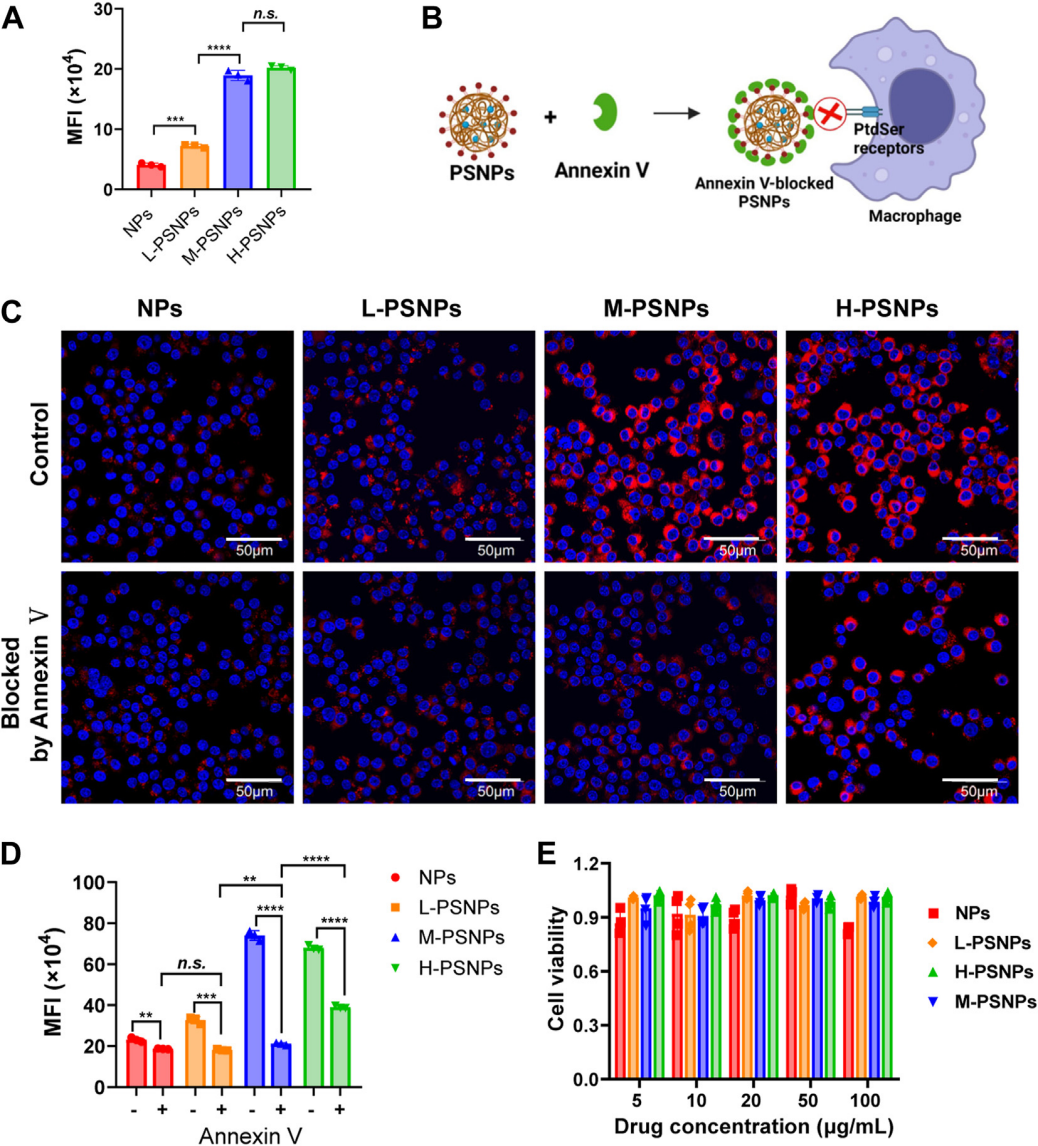
A: A flow cytometer was used to determine the MFI of macrophages after administration of DiO-loaded nanocarriers either in the presence or absence of LPS treatment (n = 3). B: Experimental setup. Annexin Ⅴ was used to evaluate the effect of PtdSer decoration on macrophage phagocytosis of PSNPs. C: The representative confocal images of macrophages after administration of DiI-loaded nanocarriers either in the presence or absence of annexin Ⅴ pretreatment. D: A flow cytometer was used to determine the MFI of macrophages after phagocytosis of nanocarriers that were pretreated with or without annexin Ⅴ (n = 3). E: CCK-8 assay was used to determine the cell viability of macrophages when exposed to DEM-loaded NPs or PSNPs (n = 4). CCK-8, cell counting kit-8; DEM, dexamethasone; DiI, 1,1′-dioctadecyl-3,3,3′,3′- tetramethylindocarbocyanine perchlorate; DiO, 3,3′-dioctadecyloxacarbocyanine perchlorate; LPS, lipopolysaccharide; MFI, mean fluorescence intensity; PtdSer, phosphatidylserine; PSNP, PtdSer-decorated PLGA nanoparticle; NP, nanoparticle.

Next, we aimed to determine whether the decoration of nanocarriers with PtdSer could help promote their phagocytosis. Macrophage phagocytosis of nanocarriers was investigated under LPS-induced inflammatory conditions. As the PtdSer concentration increased from 0% (NPs) to 2% (L-PSNPs) and then to 5% (M-PSNPs), the DiD fluorescence intensity of macrophages gradually increased, revealing that PtdSer decoration enhanced the macrophage phagocytosis of NPs (Fig. 3A). Interestingly, the fluorescence intensity of macrophages treated with H-PSNPs (PtdSer concentration of 10%) did not increase significantly compared to M-PSNPs (Fig. 3A). It is possible that the amount of PtdSer on M-PSNPs reached the saturation level of PtdSer receptors expressed on macrophages and continuing to increase the PtdSer density had little impact on phagocytosis (25).

To further verify the role of PtdSer in the macrophage phagocytosis of NPs, annexin Ⅴ was incubated with PSNPs before administration to macrophages, to block PtdSer on the PSNPs through specific binding (Fig. 3B). Confocal imaging and flow cytometry showed that the intracellular fluorescence intensity of macrophages significantly decreased after blocking the PtdSer of PSNPs, revealing that the recognition and uptake of PSNPs by macrophages was mediated by the interaction between PtdSer and recognition receptors on macrophages (Fig. 3C, D).

Last, the CCK-8 assay result showed that the cell viability did not change obviously after 24 h treatment with NPs and different PSNPs when compared to control cells (Fig. 3E), which indicated that these nanocarriers had no obvious cytotoxicity. In summary, PtdSer decoration with suitable density enhanced the phagocytosis of NPs by macrophages, which is crucial for macrophage targeting delivery in the context of ALI. According to the experimental results listed above, PSNPs with moderate PtdSer decoration density were selected and used for the following study.

### Monitoring of intracellular trafficking and disintegration in macrophages

**Fig. 4:**
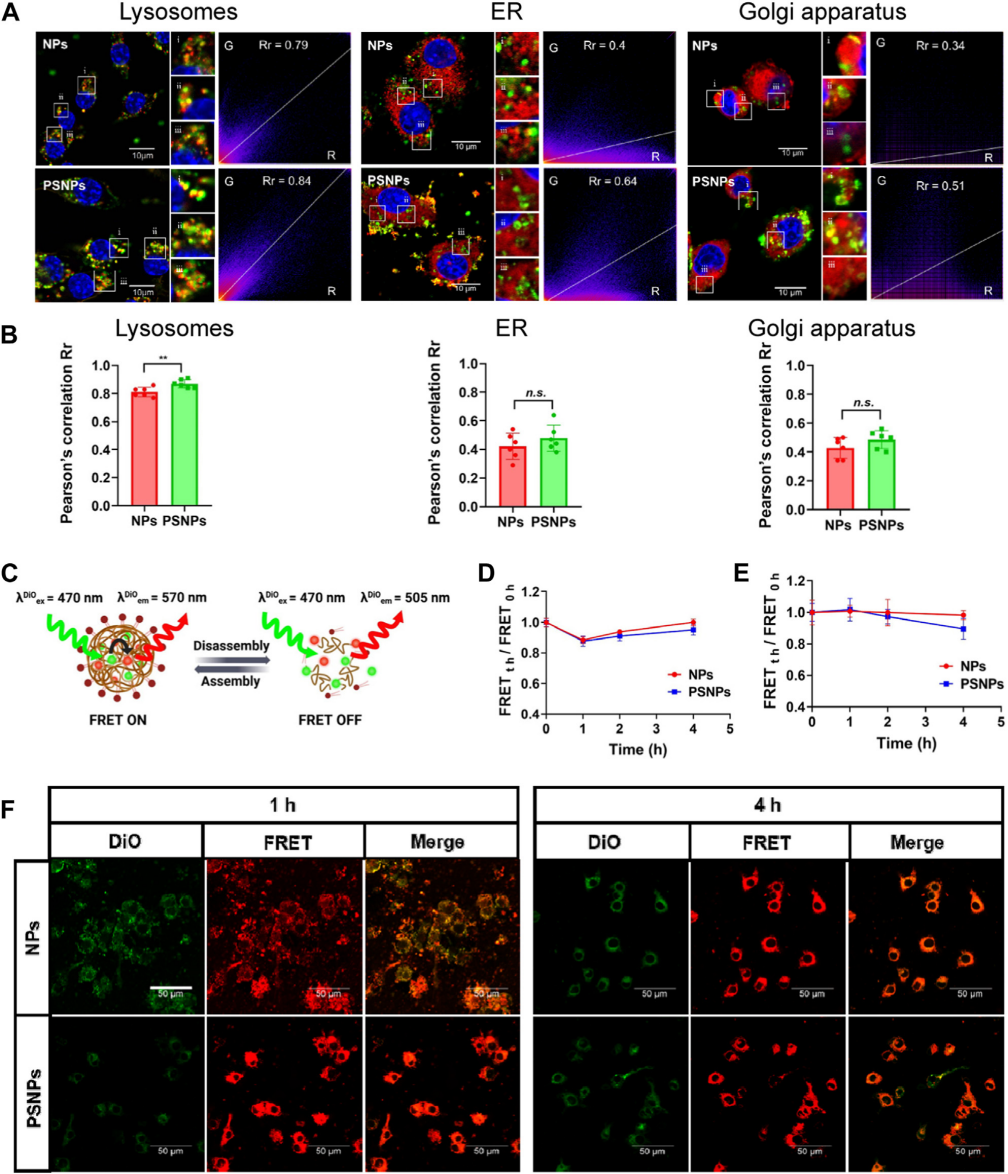
A: Confocal imaging showing the colocalization of nanocarriers with organelles (lysosomes, ER, and Golgi apparatus) in macrophages after phagocytosis; B: Pearson’s correlation Rr values were calculated by ImageJ software (n = 6, ∗∗*P* < 0.01, n.s., not significant.). C: Schematic diagram showing the FRET ON/OFF switch between DiO and DiI in nanoparticles during assembly and disassembly. D,E: The FRET ratio of nanoparticles was calculated when exposed to SLF and macrophages (n = 3). F: The representative confocal images show the changes in the FRET signals in macrophages at 1 h or 4 h after nanoparticle internalization. DiI, 1,1′-dioctadecyl-3,3,3′,3′- tetramethylindocarbocyanine perchlorate; DiO, 3,3′-dioctadecyloxacarbocyanine perchlorate; ER, endoplasmic reticulum; FRET, Förster resonance energy transfer; SLF, simulated lung fluid.

To study the intracellular trafficking pathway of PSNPs after macrophage phagocytosis, the colocalization of nanocarriers with organelles was investigated. The results of confocal imaging showed that both the NPs and PSNPs were colocalized well with lysosomes but relatively poorly with the ER and Golgi apparatus (Fig. 4A). The Pearson’s correlation Rr values showed that NPs and PSNPs had high Rr values of greater than 0.8 when colocalized with lysosomes, while the Rr values of the ER and Golgi apparatus were less than 0.5 (Fig. 4B). This result indicated that the PSNPs were mainly transported through the lysosome pathway rather than the ER/Golgi apparatus pathway.

To investigate the degradation of the nanocarriers, FRET was used to monitor the integrity of nanocarriers in the SLFs and macrophages, respectively. FRET can reflect the integrity of nanocarriers given distance-sensitive properties. Energy transfer occurs in nanocarriers when the donor fluorophore has sufficient emission spectrum overlap with the excitation spectrum of the acceptor, and the distance bettween these two fluorophores is less than 10 nm (26). In this study, FRET signals were acquired by capsulizing donor fluorophore (DiO) and acceptor fluorophore (DiI) in the hydrophobic core of PLGA NPs. The occurrence of FRET was detected by recording the emission wavelength of the acceptor fluorophore when exciting the donor fluorophore at a wavelength of 470 nm (Fig. 4C and supplemental Fig. S2). The FRET ratio did not change when the nanocarriers were incubated with SLFs for 4 h (Fig. 4D), indicating that most of the nanocarriers in SLFs were intact before macrophage uptake.

After macrophage phagocytosis, the FRET ratio of the PSNPs gradually decreased along with time. The value of FRET_t h_/FRET_0 h_ decreased from 1 to 0.89 at 4 h, revealing the degradation of the nanocarriers (Fig. 4E). Confocal imaging also showed that the donor green fluorescence of DiO became brighter as time progressed, whereas the red fluorescence of the acceptor fluorophore became weaker (Fig. 4F). In summary, these findings indicated that the PSNPs disintegrated gradually and released the drug within cells with increasing time, and this disintegration process occurred mainly in the lysosomes.

### PSNPs@DEM have dual anti-inflammatory efficacy on LPS-activated macrophages

**Fig. 5:**
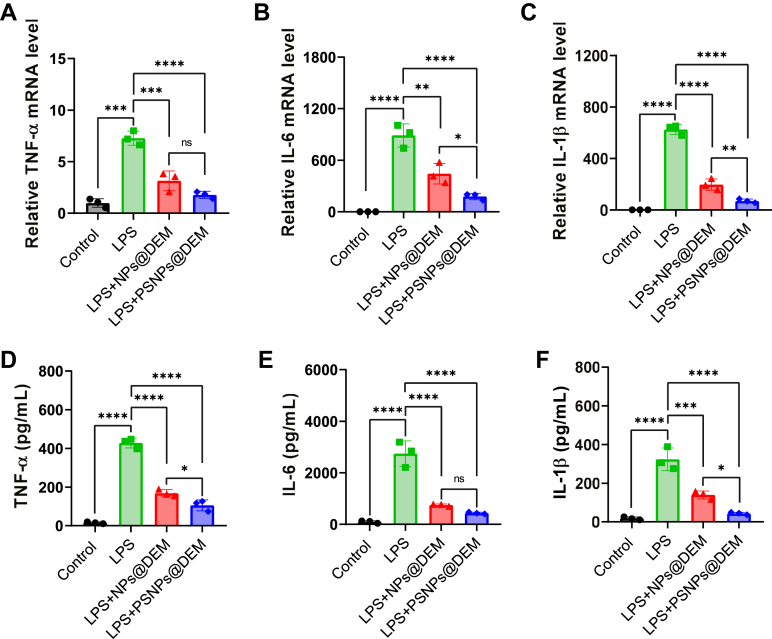
A-C: Relative TNF-α, IL-6, and IL-1β mRNA transcription levels in RAW 264.7 cells under different treatments were quantified by RT-qPCR (n = 3). D-F: The levels of cytokines in the culture supernatant from RAW 264.7 cells treated with LPS and different nanocarriers were analyzed by ELISA (n = 3). ∗*P* < 0.05, ∗∗*P* < 0.01, ∗∗∗*P* < 0.001, and ∗∗∗∗*P* < 0.0001; n.s., not significant. LPS, lipopolysaccharide; qPCR, quantitative real-time PCR.

The pathogenesis of ALI is mediated and featured by increased levels of proinflammatory cytokines (27). PtdSer has been proven to help resolve inflammation (28), and its decoration also has been shown to potentiate the anti-inflammatory effects of nanocarriers (29, 30, 31). First, the results of PCR showed that the empty PSNPs without DEM reduced inflammation in LPS-activated macrophages compared to the empty NPs, verifying the anti-inflammatory functions of PtdSer decoration (supplemental Fig. S3). Moreover, PSNPs@DEM showed superior inhibition on the mRNA expression of inflammatory cytokines (TNF-α, IL-6, and IL-1β) in LPS-activated macrophages than that of the NPs@DEM (Fig. 5A–C), which could be explained by the PtdSer decoration-mediated anti-inflammatory effect and the increased phagocytosis of NPs by macrophages. The results of the ELISA test confirmed these findings (Fig. 5D–F). Therefore, PSNPs@DEM have superior anti-inflammatory function via PtdSer-mediated anti-inflammation and accumulation in macrophages.

### PtdSer decoration enhanced lung macrophage targeting in vivo

**Fig. 6:**
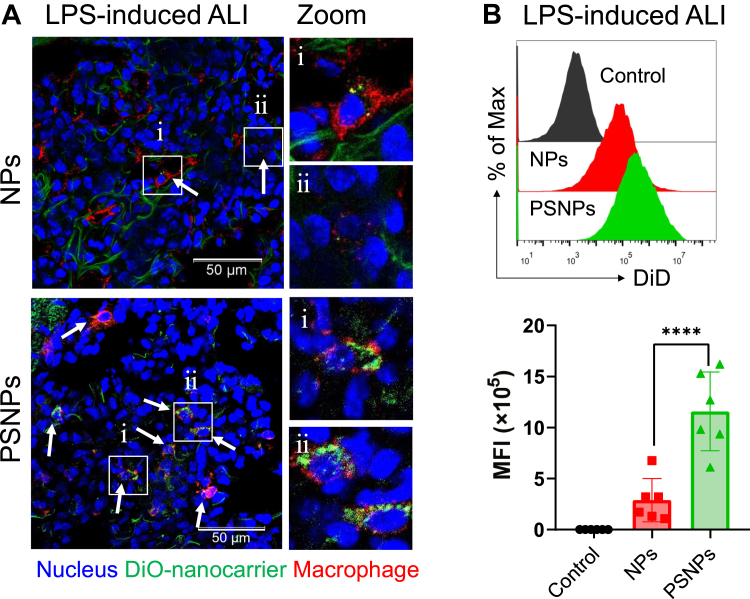
A: The representative immunofluorescence images of lung tissues from LPS-induced male ALI model mice after intratracheal administration of nanocarriers. (The scale bar is 50 μm). B: Eighteen male mice were randomly divided into three groups, including LPS-induced ALI (control group), ALI+NPs@DiD (NPs group), and ALI+PSNPs@DiD (PSNPs group). Flow cytometer showing the phagocytosis of DiD-labeled nanocarriers by F4/80-positive macrophages in the lung of male ALI mice. The dots represent data from individual male mice (n = 6). ∗*P* < 0.05, ∗∗*P* < 0.01, ∗∗∗*P* < 0.001; and ∗∗∗∗*P* < 0.0001; n.s., not significant. ALI, acute lung injury; LPS, lipopolysaccharide; DiD, 1,1′-dioctadecyl-3,3,3′,3′-tetramethylindodicarbocyanine, 4-chlorobenzenesulfonate salt; NP, nanoparticle; PSNP, PtdSer-decorated PLGA nanoparticle.

Next, we investigated whether PtdSer decoration could aid the recognition and targeting of NPs by lung macrophages in vivo. Before the experiment, the safety of the nanocarriers was confirmed by the hemolysis study (supplemental Fig. S4). Immunofluorescence imaging demonstrated that PSNPs were phagocytized by lung macrophages in an LPS-induced ALI mouse model (Fig. 6A). Moreover, we labeled the macrophages in the single-cell suspension after lung digestion with FITC-conjugated F4/80 antibodies and analyzed the fluorescence intensity of DiD in F4/80^+^ cells by flow cytometry. The result showed that the DiD fluorescence intensity of lung macrophages in PSNPs-treated mice was higher than that of cells in the NPs-treated mice (Fig. 6B). These results indicated that PtdSer decoration facilitated the recognition and targeting of NPs by lung macrophages in vivo.

### PSNPs@DEM attenuates lung injury by reducing inflammation in macrophages in situ

**Fig. 7:**
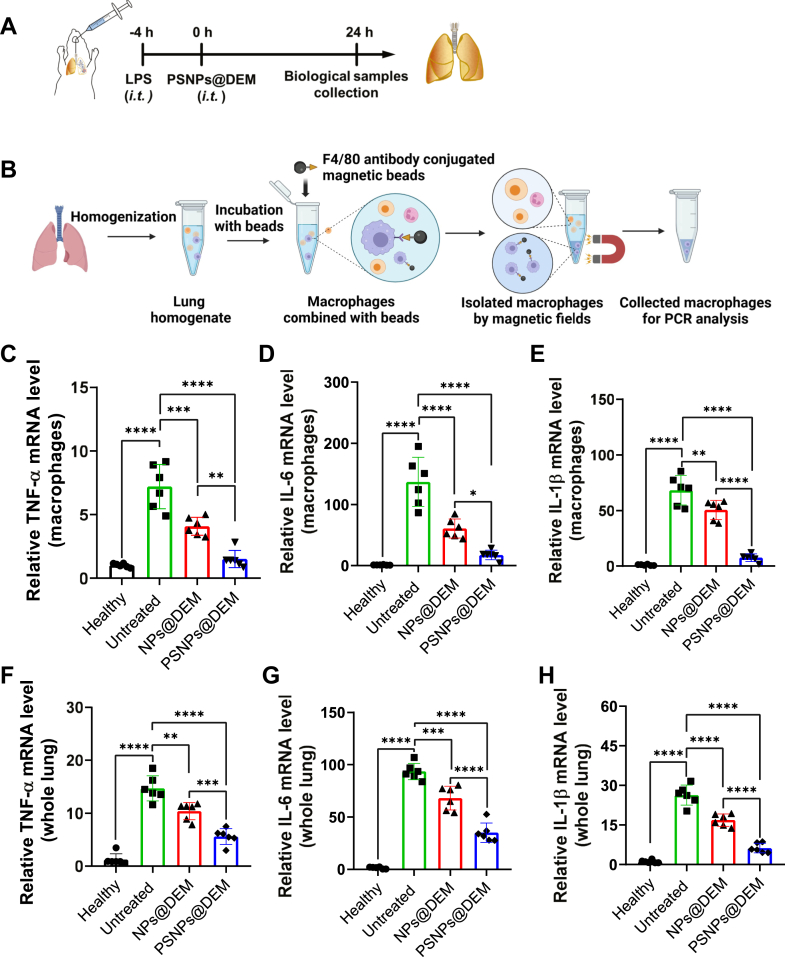
A: Experimental schedule of the ALI model establishment and treatment. B: The schematic diagram shows the isolation of lung macrophages by using magnetic-activated cell sorting beads. Twenty-four male mice were randomly divided into four groups, including healthy group, LPS-induced ALI (untreated group), ALI+NPs@DEM (NPs@DEM group), and ALI+PSNPs@DEM (PSNPs@DEM group). Relative mRNA transcription levels of TNF-α, IL-6, and IL-1β in the isolated lung macrophages (C–E) and the harvested lung tissues (F–H). The dots represent data from individual male mice (n = 6). ALI, acute lung injury; LPS, lipopolysaccharide NP@DEM, nanoparticle loaded with dexamethasone; PSNP@DEM, dexamethasone-loaded PtdSer-decorated PLGA nanoparticle.

Macrophages are the main origin of proinflammatory cytokines in lung tissues of ALI. After LPS treatment and PSNP@DEM administration (Fig. 7A), the macrophages in inflamed lungs were isolated by magnetic beads and analyzed by qPCR (Fig. 7B). We found that LPS treatment significantly elevated the mRNA expression of TNF-α, IL-6, and IL-1β in lung macrophages, and both nanocarriers loaded with DEM reduced inflammation within activated macrophages. Importantly, PSNPs@DEM exhibited higher anti-inflammatory efficacy for lung macrophages than that of the NPs@DEM (Fig. 7C–E). Moreover, we also found that PSNPs@DEM markedly decreased the mRNA expression of TNF-α, IL-6, and IL-1β in the whole lung tissues of the ALI mouse model than that of the NPs@DEM (Fig. 7F–H and supplemental Fig. S5A–C), which is an inflammation cascade after macrophage activation. These findings demonstrated that targeting delivery of drugs to lung macrophages has superior anti-inflammatory efficiency.

**Fig. 8:**
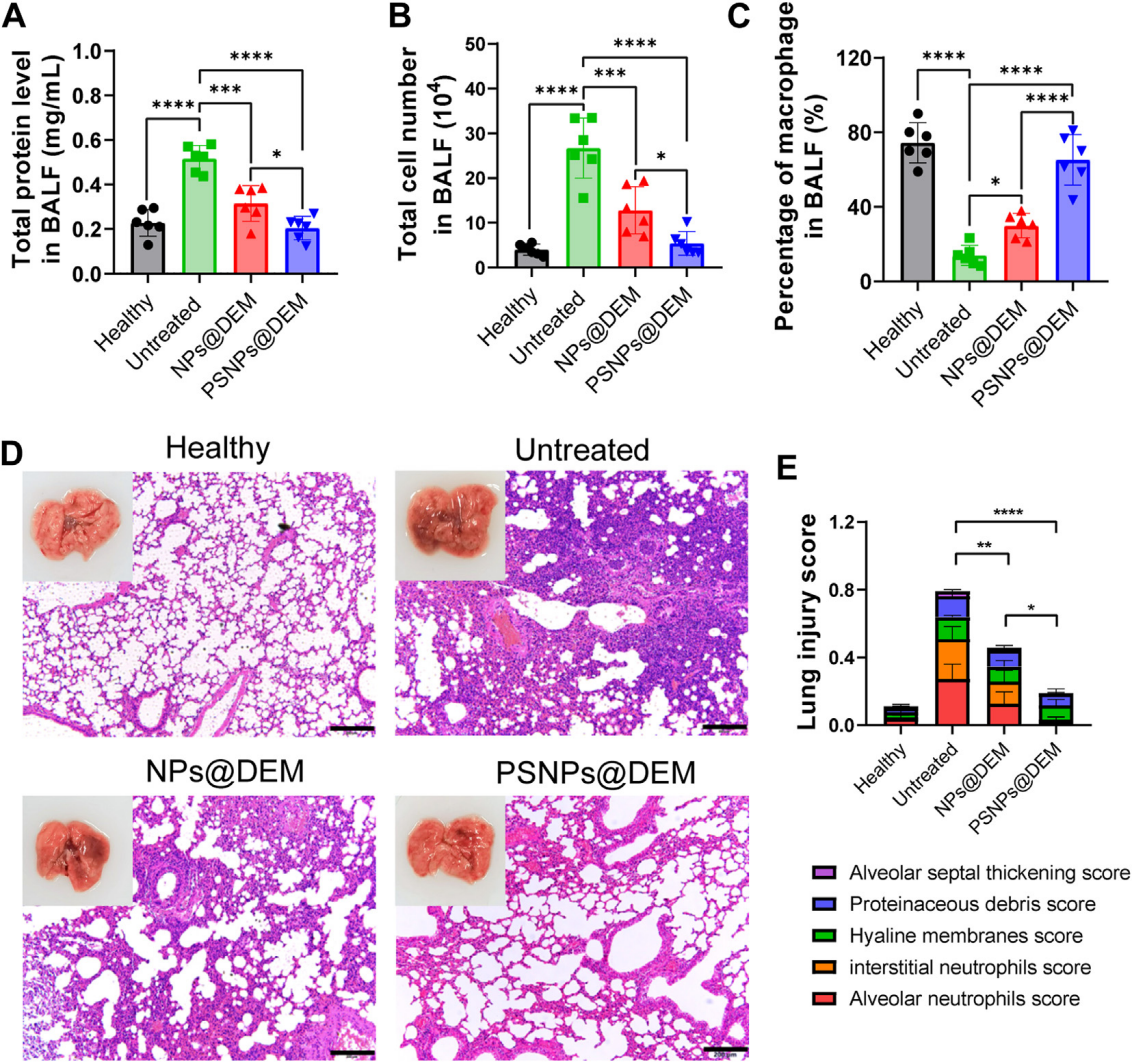
A: The total protein levels, (B) total cell number and, and (C) percentage of macrophages in BALF after treatment in an LPS-induced ALI mouse model (n = 6). Twenty-four male mice were randomly divided into four groups, including healthy group, ALI (untreated group), ALI+NPs@DEM (NPs@DEM group), and ALI+PSNPs@DEM (PSNPs@DEM group). D: The histological images of H&E-stained lung sections (scale bars = 200 μm). E: The total lung injury score was obtained from five pathophysiological features. The dots represent data from individual male mice (n = 6). ∗*P* < 0.05, ∗∗*P* < 0.01, ∗∗∗*P* < 0.001, and ∗∗∗∗*P* < 0.0001; n.s., not significant. ALI, acute lung injury; BALF, bronchoalveolar lavage fluid; LPS, lipopolysaccharide; NP@DEM, nanoparticle loaded with dexamethasone; PSNP@DEM, dexamethasone-loaded PtdSer-decorated PLGA nanoparticle.

Next, we evaluated the therapeutic effects of PSNPs@DEM on ALI. Excessive infiltration of protein-rich edema fluid and immune cells from the blood vessels to the alveolar region leads to an increase in protein concentration and total cell number in BALF under ALI conditions (32). Our results showed that the protein level and total cell number in the BALF were significantly increased in the lungs of the ALI group. Treatment with PSNPs@DEM showed a remarkable reduction in proteins and cell infiltration in lungs than did the untreated group (Fig. 8A, B). The percentages of macrophages in BALF were also better restored in the PSNP@DEM group because of reduced cell infiltration (Fig. 8C).

ALI is featured by the presence of excessive pulmonary edema and thickened alveolar walls. Compared with the untreated ALI mice, pathological changes of lungs in the PSNP@DEM-treated mice were attenuated, and the infiltration of cells in alveoli was also decreased (Fig. 8D). The severity of ALI was then assessed by scoring the five histological features of lungs (supplemental Fig. S6), including interstitial neutrophils, alveolar neutrophils, hyaline membranes, alveolar septal thickening, and proteinaceous debris (24). The assessed results demonstrated that PSNPs@DEM were able to reduce the overall lung injury score (Fig. 8E). The results showed that targeting delivery of PSNPs@DEM in lung macrophages effectively reduced inflammation in inflamed lungs and alleviated ALI syndromes.

## Discussion

In recent decades, the critical role of lung macrophages in ALI has been extensively verified (7), and lung macrophage–targeted drug delivery systems are regarded as a promising therapeutic strategy (10). Inspired by the process of apoptotic cell clearance by macrophages, we developed an apoptotic cell-mimicry NP based on a PtdSer decoration strategy, for the targeting delivery of anti-inflammatory drugs to lung macrophages to alleviate ALI. Several studies have shown that PtdSer of apoptotic cells is involved in driving anti-inflammatory activities within macrophages (28). In the present study, we found that empty PtdSer-decorated delivery platform alone reduced inflammation in LPS-activated macrophages compared to the nondecorated NPs, which is consistent with several previous studies that the decoration with PtdSer enhances the anti-inflammatory effects of nanocarriers (29, 30, 31).

Furthermore, exposure of macrophages to inflammatory stimulus leads to impaired phagocytosis (33, 34, 35), which is also reported in various inflammatory lung diseases (36). The PtdSer on the surface of apoptotic cells aids in macrophage binding and recognition (15, 16). Our result demonstrated that PtdSer decoration on the surface of NPs at a suitable density enhanced the phagocytosis of NPs by inflammatory macrophages, which is crucial for macrophage targeting delivery in inflamed lungs.

Although PtdSer modification helps improve the recognition of phagocytic NPs by macrophages, attention should also be paid to the possible clotting cascade caused by highly PtdSer exposure on the vascular epithelium (37, 38). On the way toward the nanocarrier testing, we found the H-PSNPs (PtdSer concentration of 10%) caused obvious red blood cell accumulation in the mouse lung. However, no such issue was observed in the lungs of L-PSNPs (PtdSer concentration of 2%) and M-PSNPs (PtdSer concentration of 5%) groups. We speculated that this clotting issue was attributed to the different amounts of PtdSer exposure in vessels. However, the underlying mechanism is unclear. More comprehensive and in-depth studies are needed to explore the link between PtdSer levels and their potential clotting mechanism in the future.

Given that clinical ALI patients typically require mechanical ventilation due to respiratory failure, the administration route of NPs employed in animal studies (intratracheal instillation) is not suitable for clinical patients. Aerosol inhalation generated by the nebulizer might be a potential administration route for PtdSer decorated nanocarriers in the clinic. This approach aligns with clinical respiratory support practices, allowing simultaneous integration with ventilator circuits. For ALI patients receiving aerosol nebulization in conjunction with mechanical ventilation, it is important to ensure the proper ventilator settings, for instance, higher tidal volume and/or recruitment maneuver for extensive drug distribution (39). This provides an exploration direction for the clinical application of PSNPs in the future.

## Conclusions

Here, we found PtdSer decoration enhanced the phagocytosis of NPs by macrophage, and this process was affected by PtdSer decoration densities. Second, empty PSNPs alone reduced inflammation in macrophages compared to noncoated NPs. Last, we demonstrate that PSNPs successfully aid to deliver a corticosteroid drug (DEM) to the lung macrophages after pulmonary administration and to inhibit macrophage inflammation in an LPS-induced ALI model. Collectively, these findings demonstrated that PtdSer decoration not only endows the anti-inflammatory function to the delivery platform but also potentiates its macrophage targeting in the inflamed microenvironment, which might be a promising therapeutic strategy to alleviate ALI syndromes without off-target effects.

## Data availability

All data are included in the article.

## Supplemental data

This article contains supplemental data.

## Conflicts of interest

The authors declare that they have no conflicts of interest with the contents of this article.

## Supplementary data

Revised supplementary materials
