# Supplementary material for: Phosphatidylserine-decorated delivery platform helps alleviate acute lung injury via potentiating macrophage targeting
Source: J Lipid Res. 2025 Apr 10;66(5):100799. doi: 10.1016/j.jlr.2025.100799 (PMC12127575; doi:10.1016/j.jlr.2025.100799)
Supplement: Revised supplementary materials [file mmc1.docx]

**Supplementary materials**

**Phosphatidylserine decorated delivery platform helps alleviate acute lung injury via potentiating macrophage targeting**

Yue Li^2†^, Hu Li^1†^, Zhiwei Hu^3^, Yayue Zhang^1^, Xuran Ding^1^, Xinjie Huang^1^, Yabing Hua^1^, Lin Sun^4^, Ye Li^5^, Ziming Zhao^1*^, Yuan He^1*^

^1^ School of Pharmacy, Xuzhou Medical University, Xuzhou, Jiangsu 221004, China;

^2^ School of Medical Technology, Xuzhou Medical University, Xuzhou, Jiangsu 221004, China;

^3^ The Second Clinical Medical School, Xuzhou Medical University, Xuzhou, Jiangsu 221004, China;

^4^ Department of Zhuhai Campus of Zunyi Medical University, Zhuhai 519041, China

^5^ The Third Affiliated Hospital of Sun Yat-sen University, Guangzhou, Guangdong, China

^†^These authors have contributed equally to this work and share first authorship

^*^These authors have contributed equally to this work and share last authorship

**Corresponding author:**

Prof. Yuan He, Ph.D., E-mail: yuanhe@xzhmu.edu.cn

Prof. Ziming Zhao, Ph.D., E-mail: zmzhao@xzhmu.edu.cn

**1. Primers**

**Table S1** The sequences of primers used in this study.

| Primers | Sequences |
| --- | --- |
| β-actin | 5′-GTGACGTTGACATCCGTAAAGA-3′ (forward)  5′-GCCGGACTCATCGTACTCC-3′ (reverse) |
| TNF-α | 5′-CAGGCGGTGCCTATGTCTC-3′(forward)  5′-CGATCACCCCGAAGTTCAGTAG-3′ (reverse) |
| IL-6 | 5′-TACCACTTCACAAGTCGGAGGC-3′ (forward)  5′-CTGCAAGTGCATCATCGTTGTTC-3′ (reverse) |
| IL-1β | 5′-GAAATGCCACCTTTTGACAGTG-3′ (forward)  5′-TGGATGCTCTCATCAGGACAG-3′ (reverse) |

**2. Characterization of nanocarriers with different PtdSer densities**

**Table S2** Characterization of NPs@DEM and PSNPs@DEM with different PtdSer densities (n=3).

| Samples | Size (nm) | PDI | Zeta potential (mV) | Encapsulation efficiency (%) | Drug loading efficiency (%) |
| --- | --- | --- | --- | --- | --- |
| NPs@DEM | 213.13 ± 12.61 | 0.11 ± 0.02 | -13.93 ± 2.09 | 52.29 ± 4.27 | 17.43 ± 1.42 |
| L-PSNPs@DEM | 202.9 ± 6.68 | 0.18 ± 0.02 | -19.84 ± 2.29 | 53.05 ± 3.83 | 17.45 ± 1.26 |
| M-PSNPs@DEM | 208.32 ± 7.34 | 0.12 ± 0.06 | -22.73 ± 1.14 | 51.39 ± 2.72 | 16.59 ± 0.88 |
| H-PSNPs@DEM | 183.62 ± 11.44 | 0.13 ± 0.05 | -23.85 ± 2.56 | 52.87 ± 4.07 | 16.52 ± 1.27 |

**3. Characterization of NPs and PSNPs**

**
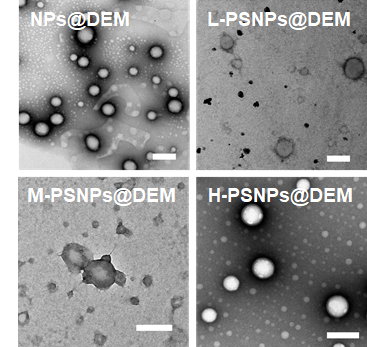
**

**Figure S1.** TEM images of the prepared nanoparticles (the scale bar is 200 nm);

**4. Validation of FRET signals in NPs and PSNPs**


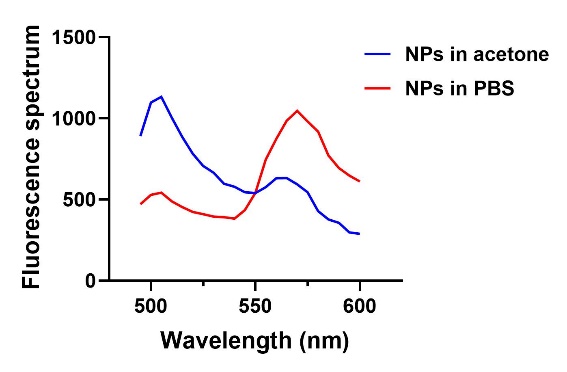

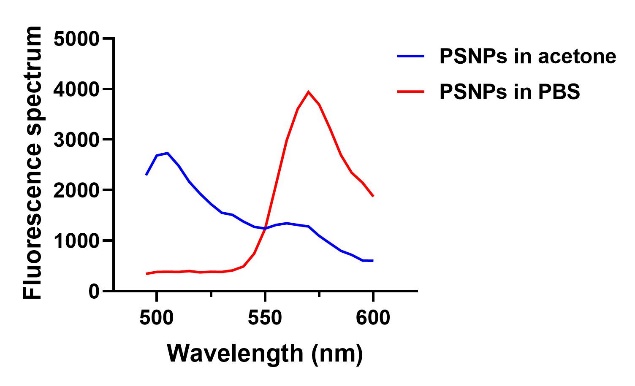


**Figure S2.** The fluorescence spectrum of DiO/DiI-loaded NPs and DiO/DiI-loaded PSNPs in acetone and PBS buffer.

**5. Anti-inflammatory effects of empty PSNPs in vitro**


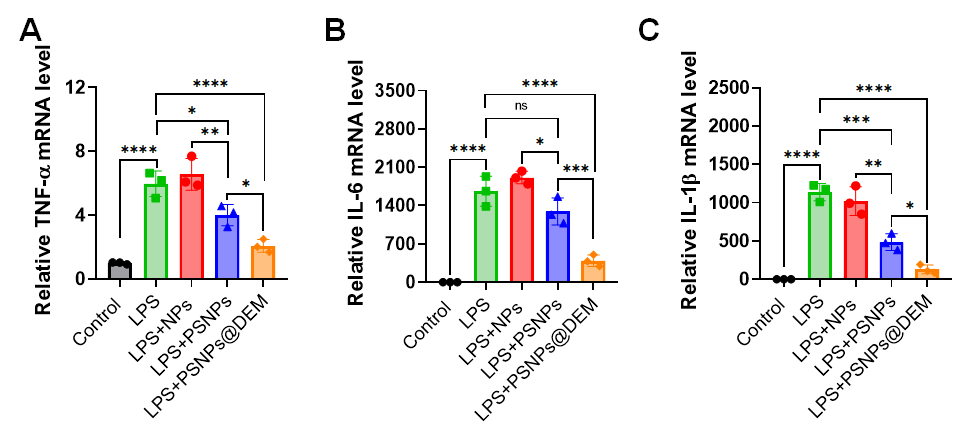


**Figure S3.** (**A, B, C**) Relative TNF-α, IL-6, and IL-1β mRNA transcription levels in RAW 264.7 cells under different treatments was quantified by RT-qPCR (n=3). **p* < 0.05, ***p* < 0.01, ****p* < 0.001, *****p* < 0.0001; *n.s.*, not significant.

**6. Hemolysis assay**

Peripheral blood was collected and diluted to 1/10 of their volume with PBS, and then 0.3 mL of the solution was added into a 1.5 mL centrifuge tube, followed by centrifuging at 2000 g for 5 min to remove the residual PBS. Next, PSNPs in 1.0 mL of PBS were added to the precipitate and gently mixed on a horizontal shaker at a speed of 100 rpm at 37 °C. The groups treated with 1 × PBS or Triton-X-100 were used as negative and positive controls, respectively. Two hours later, the samples were centrifuged at 2000 g for 5 min, and the absorbance of the supernatants at 541 nm was measured by a microplate reader (BioTek Instruments, USA). Hemolysis percentages of the PSNPs were calculated with the following equation: Hemolysis % = (A_sample_-A(-)_control_)/(A(+)_control_- A(-)_control_). A is the absorbance value.

According to the result, hemolysis phenomenon could hardly be observed by visual inspection in PSNPs. Then the percentage of hemolysis was calculated by microplate reader to assess the hemolysis activity. Result showed that PSNPs caused about 3 % hemolysis, and the difference between PSNPs groups with PBS negative group was not significant (p > 0.05). On the other hand, Triton X-100 which was used as the positive control group caused about 100 % hemolysis (Fig. S4).


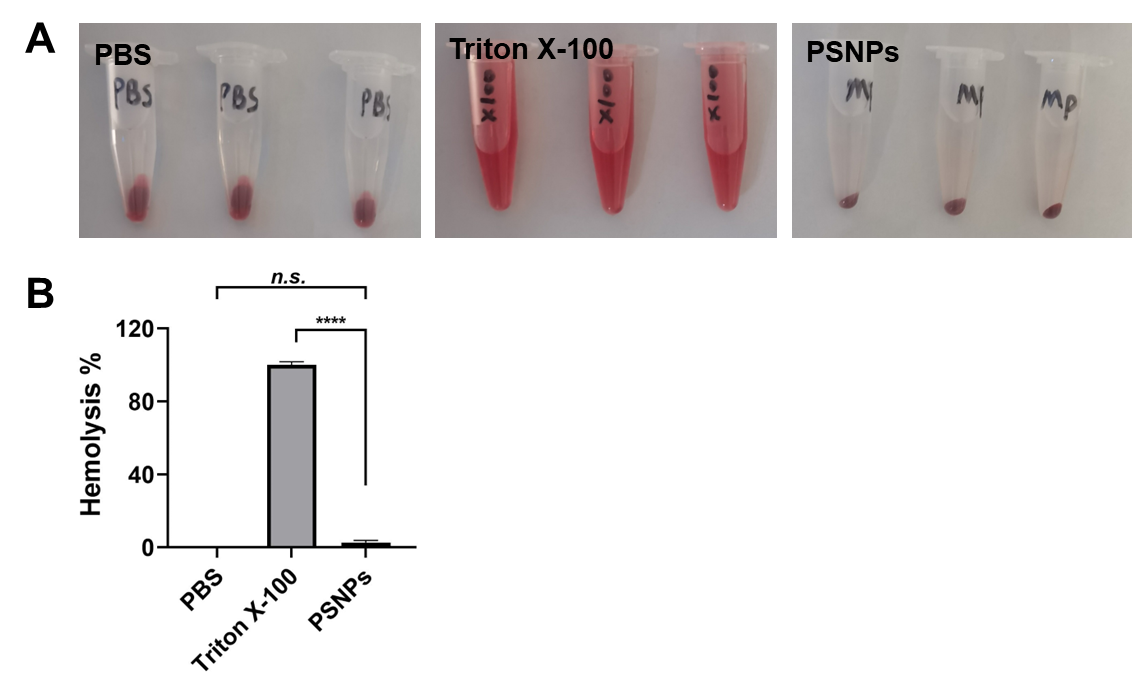


**Figure S4.** (A) Visual inspection of the tubes containing diluted blood after exposure to PSNPs for 2 h after centrifugation. PBS and Triton-X-100 were respectively used as negative control and positive control. (B) Hemolysis percentages after exposure to PSNPs (n = 3). *****p* < 0.0001, *n.s.*, not significant.

**7. The applicability of PSNPs@DEM in the female ALI mice**


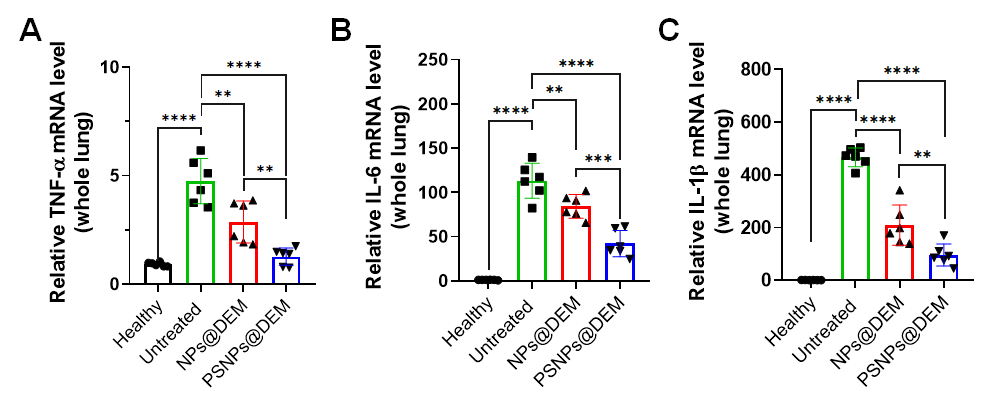


**Figure S5.** Twenty-four female mice were randomly divided into four groups, including healthy group, LPS-induced ALI (untreated group), ALI + NPs@DEM (NPs@DEM group), and ALI + PSNPs@DEM (PSNPs@DEM group). Relative mRNA transcription levels of TNF-α, IL-6, and IL-1β in the harvested lung tissues (**A-C**). The dots represent data from individual female mice (n = 6). **p* < 0.05, ***p* < 0.01, ****p* < 0.001, *****p* < 0.0001; *n.s.*, not significant.

**8. Pathological Scores of ALI mouse model**


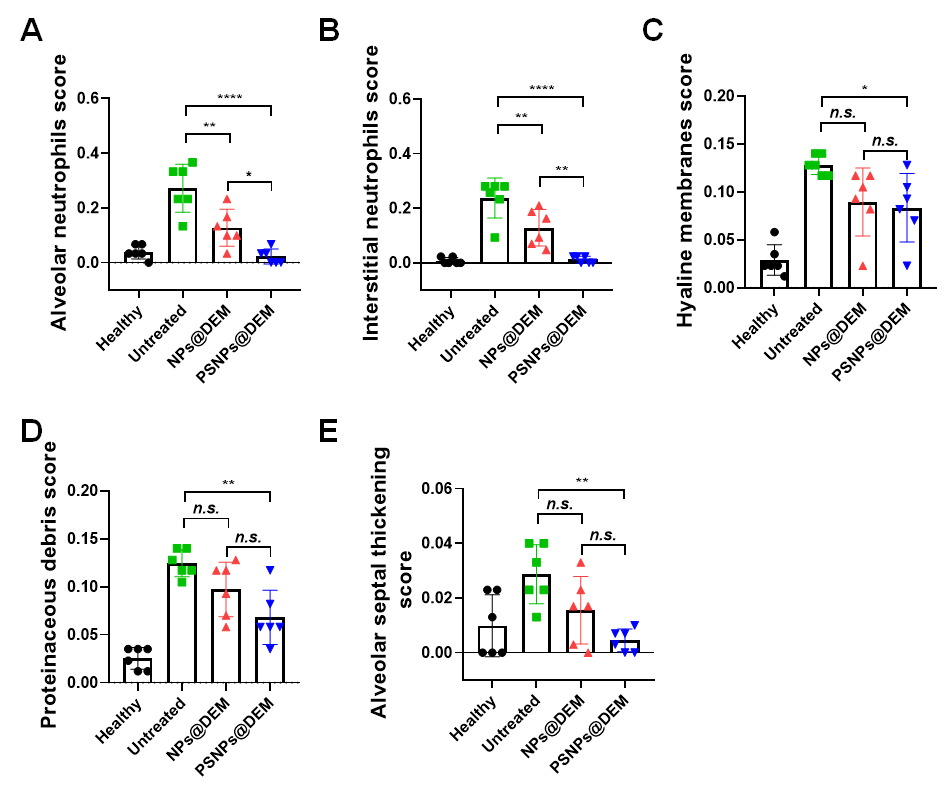


**Figure** **S6.** Scores of pathological features based on the histological images. (**A**) Alveolar neutrophils score; (**B**) interstitial neutrophils score; (**C**) hyaline membranes score; (**D**) proteinaceous debris score; (**E**) alveolar septal thickening score. Twenty-four male mice were randomly divided into four groups, including healthy group, ALI (untreated group), ALI + NPs@DEM (NPs@DEM group), and ALI + PSNPs@DEM (PSNPs@DEM group). The dots represent data from individual male mice (n = 6). **p* < 0.05, ***p* < 0.01, ****p* < 0.001, *****p* < 0.0001; *n.s.*, not significant.
